# Reproductive Health Assessment of Female Elephants in North American Zoos and Association of Husbandry Practices with Reproductive Dysfunction in African Elephants (*Loxodonta africana*)

**DOI:** 10.1371/journal.pone.0145673

**Published:** 2016-07-14

**Authors:** Janine L. Brown, Stephen Paris, Natalia A. Prado-Oviedo, Cheryl L. Meehan, Jennifer N. Hogan, Kari A. Morfeld, Kathy Carlstead

**Affiliations:** 1 Center for Species Survival, Smithsonian Conservation Biology Institute, Front Royal, Virginia, United States of America; 2 AWARE Institute, Portland, Oregon, United States of America; 3 Lincoln Children’s Zoo, Lincoln, Nebraska, United States of America; 4 Portland, OR, United States of America; University of Tasmania, AUSTRALIA

## Abstract

As part of a multi-institutional study of zoo elephant welfare, we evaluated female elephants managed by zoos accredited by the Association of Zoos and Aquariums and applied epidemiological methods to determine what factors in the zoo environment are associated with reproductive problems, including ovarian acyclicity and hyperprolactinemia. Bi-weekly blood samples were collected from 95 African (*Loxodonta africana*) and 75 Asian (*Elephas maximus*) (8–55 years of age) elephants over a 12-month period for analysis of serum progestogens and prolactin. Females were categorized as normal cycling (regular 13- to 17-week cycles), irregular cycling (cycles longer or shorter than normal) or acyclic (baseline progestogens, <0.1 ng/ml throughout), and having Low/Normal (<14 or 18 ng/ml) or High (≥14 or 18 ng/ml) prolactin for Asian and African elephants, respectively. Rates of normal cycling, acyclicity and irregular cycling were 73.2, 22.5 and 4.2% for Asian, and 48.4, 37.9 and 13.7% for African elephants, respectively, all of which differed between species (P < 0.05). For African elephants, univariate assessment found that social isolation decreased and higher enrichment diversity increased the chance a female would cycle normally. The strongest multi-variable models included Age (positive) and Enrichment Diversity (negative) as important factors of acyclicity among African elephants. The Asian elephant data set was not robust enough to support multi-variable analyses of cyclicity status. Additionally, only 3% of Asian elephants were found to be hyperprolactinemic as compared to 28% of Africans, so predictive analyses of prolactin status were conducted on African elephants only. The strongest multi-variable model included Age (positive), Enrichment Diversity (negative), Alternate Feeding Methods (negative) and Social Group Contact (positive) as predictors of hyperprolactinemia. In summary, the incidence of ovarian cycle problems and hyperprolactinemia predominantly affects African elephants, and increases in social stability and feeding and enrichment diversity may have positive influences on hormone status.

## Introduction

One of the key opinions in both ethical and scientific discussions of animal welfare is that animals should be maintained under conditions that support good biological functioning in the sense of health, growth and reproduction [1]. When reproductive dysfunction is apparent at the population level, it raises concerns about the potential negative impacts housing and management factors may have on the biological systems that regulate these functions [2,3]. Reproductive problems facing North American populations of Asian and African elephants are well documented and, for African elephants, a primary cause of poor reproduction is a high rate of ovarian acyclicity [4,5]. Based on the last reproductive survey of elephants in American Zoo and Aquarium (AZA)-accredited zoos, 46% of African elephant females exhibited abnormal ovarian cycles, while 31% did not cycle at all. Most importantly, the majority of ovarian cycle problems occurred in reproductive age females [6].

Despite the recognition of this problem for over two decades, the etiology remains a mystery. There is considerably more information available on what does not cause acyclicity than what does. For example, several conditions known to be associated with infertility in other species have been found not to be related to ovarian problems in African elephants, such as hyperandrogenism [7], hyperestrogenism [8], thyroid derangements, pituitary dysfunction [9,10] or elevated cortisol [10,11]. Another disproven theory is that acyclic zoo elephants have exhausted their supply of ovarian follicles; i.e., undergone a premature ‘menopause’. Non-conceptive zoo elephants have the capacity to cycle continuously, whereas free-ranging females are often pregnant or lactating and thus experience comparatively fewer cycles [4]. In most mammals, including African elephants [12], the ovarian reserve is high at birth and undergoes a steady loss through natural attrition [13], becoming depleted as reproductive senescence ensues [14]. Anti-müllerian hormone (AMH) is produced by granulosa cells and serves as a marker for the number of morphologically healthy oocytes within the follicular reserve [15,16]. But, in a study by Dow et al. [17] no differences were found between cycling and acyclic elephants in AMH concentrations, suggesting ovarian acyclicity need not be a permanent condition. This is supported by numerous examples of female elephants switching cyclicity status (cycling to acyclic, and vice versa) [4,5]. Thus, if factors contributing to ovarian cycle problems could be identified, the condition might be reversed in many individuals.

One association with ovarian acyclicity in African elephants is a hormonal imbalance–hyperprolactinemia [10,18,19]. In other species, hyperprolactinemia is associated with infertility [20–22], and in women it is the most common disorder of the hypothalamic-pituitary axis; up to 40% presenting with secondary amenorrhea are hyperprolactinemic [23,24]. The negative effects of chronic elevated prolactin secretion on reproductive function generally involve inhibition of hypothalamic GnRH release and subsequent suppression of pituitary LH and FSH secretion, resulting in anovulation [25]. Pituitary prolactin secretion is under inhibitory control by dopamine [25–27] and there is evidence the same negative feedback mechanism works in elephants, based on findings that a dopamine agonist (cabergoline) decreases and an antagonist (domperidone) increases prolactin secretion [5,28]. In addition to hypothalamic inhibition by dopamine, other factors can stimulate prolactin synthesis or have a suppressive effect on dopaminergic tone: vasoactive intestinal polypeptide, estradiol, serotonin, oxytocin, thyrotropin-releasing hormone (TSH) and vasopressin [29]. Of these, studies of estradiol and thyroid hormones (TSH, T_3_, T_4_), have found no relation to elevated prolactin in elephants [8,10]. Another cause could be stress, as prolactin is often secreted in response to physical or psychological stressors [30,31], but cortisol has not been found to be related to prolactin levels in elephants [10].

It is important to understand why so many elephant females are not cycling normally, and are developing hyperprolactinemia. Prolonged acyclicity does not typically occur in wild African elephants based on physiological studies that show females can cycle into their 50’s [32,33]. Thus, it appears to be an *ex situ* issue, although because the majority of zoos house both cycling and acyclic females [6,34], there may be multiple etiologies. For elephants, associations between specific management or social factors and reproductive status have not been identified. Thus, the objectives of this study were to: 1) assess the current ovarian cycle status of Asian and African elephants in the AZA population; 2) determine the incidence of hyperprolactinemia and its relationship to ovarian acyclicity; 3) and conduct a multi-variable analysis to determine how housing, social and management factors are related to the chance that African female elephants will cycle and have normal prolactin. We can try to develop targeted treatments for these conditions, but given the complexity of endocrine function, it is more important to identify underlying causes so that mitigating steps can be taken to prevent problems from occurring in the first place. Therefore, it would be beneficial to understand how social and environmental factors relate to prolactin secretion and reproductive status, and if husbandry changes could help elephants better cope with captive environments, similar to that described for other species [35–39].

## Materials and Methods

### Ethics Statement

All elephants included in this study were enrolled in the Using Science to Understand Zoo Elephant Welfare project [40]. Our study was authorized by the management at each participating zoo and, where applicable, was reviewed and approved by zoo research committees. In addition the study protocol was reviewed and approved by the Zoological Society of San Diego Institutional Animal Care and Use Committee (N.I.H. Assurance A3675-01; Protocol 11–203). Approval also was obtained from the Smithsonian National Zoo ACUC (#11–10).

### Animals and Blood Sample Collection

Female elephants between the ages of 8–55 years who were not currently pregnant and had not given birth between 2010 and 2012, and who did not die or experience an inter-zoo transfer in 2012, were included in the study population. However, elephants that met the above criteria were exempted from the study if they were not well conditioned to blood collection procedures. Our subject population was comprised of 75 Asian and 95 African elephant females from 66 zoos. Blood samples were collected every other week without anesthesia from either an ear or leg vein. Protocols requested blood draws to occur before 12 noon. Blood was allowed to clot at room temperature, centrifuged (~1500 g) and the serum stored frozen at -20˚C or colder until analysis.

### Hormonal Analyses

Serum progestogens were analyzed using a solid-phase I^125^ radioimmunoassay (RIA) (Seimens Medical Solutions Diagnostics, Los Angeles, CA), validated for elephants [5,10,41]. Serum prolactin was analyzed by a heterologous RIA that utilized an anti-human prolactin antisera (NIDDK-anti-hPRL-3) and ovine prolactin label and standards (NIDDK-oPRL-I-2), also validated for elephant serum [5,41,42]. Assay sensitivities (based on 90% maximum binding) were 0.05 and 5.0 ng/ml for the progesterone and prolactin RIAs, respectively. The intra- and inter-assay coefficients of variation for all assays were <15% and <10%, respectively.

### Determination of Reproductive Cyclicity and Prolactin Status

**Fig 1:**
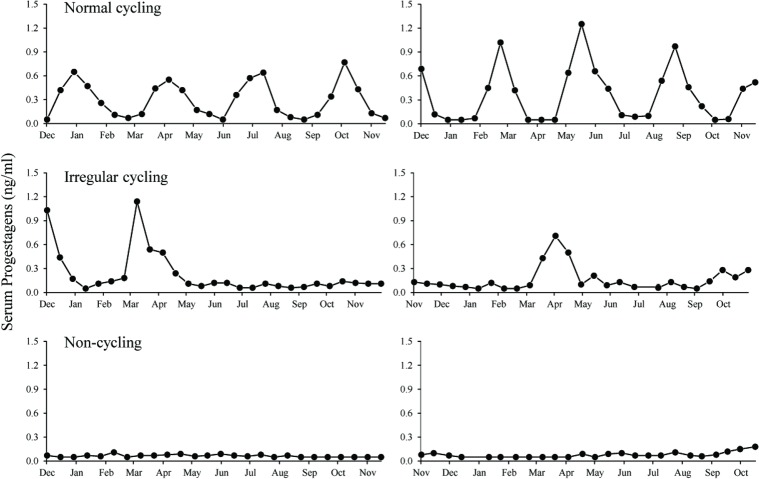
Representative serum progestogen profiles for normal cycling (upper panels), irregular cycling (middle panels) and acyclic (lower panels) female African elephants blood sampled bi-weekly.

Estrous cycle characteristics were determined based on serum progestogen levels. First, baseline values were calculated for each individual using an iterative process developed for elephants [10,43,44]. For each animal, all data points with progestogen values above the mean plus 1.5 times the standard deviation (SD) were removed and the process repeated until no values exceeding the mean + 1.5*SD remained. The remaining data points defined the baseline for that individual. Estrous cycle lengths were classified as described by Glaeser et al. [44]. The first and last week of the luteal phase were based on the following criteria: 1) the luteal phase was defined as progestogen concentrations greater than baseline for at least 2 consecutive weeks, with a duration of at least 4 weeks; 2) the follicular phase was defined as progestogen concentrations below the baseline for at least 2 consecutive weeks; 3) single point fluctuations above or below baseline were considered within the same phase as the surrounding points; 4) data points on the baseline were included in the previous phase; 5) when data were not available for a given week, and that week appeared to coincide with the start or end of a luteal phase, it was added to the luteal phase. Estrous cycle duration was calculated as the number of weeks from the first increase in serum progestogens until the next increase. Data were used to categorize the ovarian cycle status of each elephant as follows: normal cycling (regular 12- to 18-week progestogen cycles); irregular cycling (cycle durations outside ± 2*SD of the population mean [44]); or acyclic (baseline progestogens, <0.1 ng/ml, throughout) [10]. Representative profiles for cycling, irregular cycling and acyclic female elephants are shown in Fig 1.

Hyperprolactinemia for African elephants was defined as an average prolactin concentration over the 12-month sampling period of ≥18 ng/ml, similar to Prado-Oviedo et al. [8], which lacked a temporal pattern [19]. Cut-off values were calculated for Asian elephants and determined to be < or ≥14 ng/ml. Because there were only two Asian elephants with high prolactin, statistical analyses were not performed. For statistical analysis of African elephants, females with an average prolactin concentration of 18 ng/ml or greater were considered “High”, and those below 18 ng/ml were considered “Low/Normal”.

### Independent Variables

Independent variables were selected based on hypotheses regarding their potential association with ovarian cyclicity and prolactin status. Titles of each variable and their definitions are presented in Table 1. Details on the collection and calculation of demographic variables are presented in Prado-Oviedo et al. [45], housing and social variables are presented in Meehan et al. [46], and Greco et al. [47] provides background on variables related to exercise, training, feeding practices and environmental enrichment.

**Table 1:** Description of variables used in analysis of cyclicity for Asian and African elephants and hyperprolactinemia for African elephants.

Variable	Unit of Analysis	Unit	Description	Ref
*Demographics*^1^				
Age	Elephant		Age of elephant (years)	1
Origin	Elephant		Captive or wild born	1
*Space and Social*^2^				
Herd Size	Zoo		Total number of elephants at zoo	2
Animal Contact	Elephant		Maximum number of unique elephants focal animal is in contact with	2
Social Group Count	Zoo		Number of unique social groups at zoo	2
Social Group Contact	Elephant		Maximum number of unique social groups focal animal is part of	2
Space Experience			The average weighted (by percent time) size of all environments in which an elephant spent time.	
Total	Elephant	(ft^2^)	For all environment types	2
Indoor	Elephant	(ft^2^)	For indoor environments only	2
In/Out Choice	Elephant	(ft^2^)	For environments where there is a choice of indoors or outdoors	2
Outdoor	Elephant	(ft^2^)	For outdoor environments only	2
Space Experience per Elephant	Elephant		Average weighted size of each environment an elephant spends time in, divided by total number of elephants in social group using the environment at that time, weighted by the amount of time spent in each environment	2
Relative Space Experience Change	Elephant		(Total Day Space Experience—Total Night Space Experience)/(Total Day Space Experience)	2
Social Experience	Elephant		The average weighted (by percent time) size of all social groups in which an elephant spent time.	2
Relative Social Experience Change	Elephant		(Total Day Social Experience—Total Night Social Experience)/(Total Day Social Experience)	2
*Percent Time*^2^			Sum of monthly percent time spent in category, averaged over time period	
Indoor	Elephant	%	Time spent in indoor environments	2
In/Out Choice	Elephant	%	Time spent in environments with an indoor/outdoor choice	2
Outdoor	Elephant	%	Time spent in outdoor environments	2
Housed Separately	Elephant	%	Time spent housed alone	2
With Restricted Physical Access	Elephant	%	Time spent housed alone with potential for physical contact with one or more other elephants through a barrier	2
With Juveniles (<7 years old)	Elephant	%	Time spent in social groups where an elephant 7 years or younger waspresent	2
With Mixed Sex	Elephant	%	Time spent in social groups where both males and females were present	2
*Feeding*^3^				
Feed Total	Zoo		Sum of feedings during the day and night	3
Feeding Diversity	Zoo		Shannon diversity index of the number of feeding types and frequency with which each type was provided	3
Alternative Feeding Methods	Zoo		Proportion of all feedings where food was presented in a foraging device, hidden, or hung above the exhibit.	3
Feeding: Spread	Zoo		Proportion of all feedings where food was spread through the exhibit	3
*Training*^3^				
Percent Time Spent Managed	Elephant	%	Sum of percent time spent in staff-directed activities.	3
Percent Independent Time	Elephant	%	Sum of time spent outside of staff-directed activities.	3
*Exercise*, *Enrichment and Health*^3^				
Enrichment Program			Standardized Factor Score created using a polychoric PCA to examine the frequency of use of the different components of an enrichment program	3
Exercise Diversity	Zoo		Shannon diversity index of the number of exercise types and the frequency with which each type was used.	3
Enrichment Diversity	Zoo		Shannon diversity index of the number of enrichment types and frequency with which they were provided	3

See

^1^Prado et al. [45]

^2^Meehan et al. [46] and

^3^Greco et al. [47] for variable definitions and descriptions.

### Statistical Analysis

Data are presented as means ± SEM. Differences in cyclicity status and mean prolactin concentrations between species were determined using a Mixed Model ANOVA, which took into account the multiple cycles and, therefore, repeated data measures, followed by LSD post hoc tests. Pearson’s Chi Square analyses were used to test for differences in percentage data for cyclicity results between species. Due to the low frequencies of acyclicity and hyperprolactinemia among Asian elephants, no further analyses were conducted on this population.

For African elephants, separate predictive models for cyclicity and prolactin status were fitted using generalized estimating equations (GEE), which allow for repeated measurement and clustering of individual animals within zoos, and used a logistic binomial distribution. Reference values were set as Cycling = “Yes” and Prolactin Level = “Low/Normal”. An independent correlation structure was specified. Multi-variable regression models were built by first assessing individual predictors at the univariate level, and any variables that significantly predicted cyclicity or prolactin status (P < 0.15) following these assessments were retained for evaluation in the hierarchical model building process. Confounding variables (those that altered the beta values of input variables by more than 10% during bivariate analysis) were included in all models. Once a pool of viable input variables and confounders were identified, the hierarchical model building process proceeding using the forward selection approach [48]. Models reaching the multi-collinearity criteria, as defined by a variance inflation factor of greater than 10 and a condition index of greater than 30, were not considered for further analysis [48]. The forward selection of variables was continued until the addition of variables no longer resulted in significant models. Interactions were assessed after each hierarchical step and the final model was selected based on quasi-likelihood under the independence model criterion (QIC) values [49] and parameter estimates of explanatory variables. Regression coefficients were exponentiated such that results are presented as Odds Ratios (OR) to aid in interpretation. The OR represents the ratio of the odds of an outcome (acyclicity, hyperprolactinemia) occurring given a particular exposure (elephant demographic or management factors) compared to the odds of the outcome occurring given non-exposure.

Statistical analyses were conducted using SAS software, version 9.3 (PROC GENMOD, with options REPEATED, CORR = IND, DIST = BINOMIAL, LINK = LOGIT; SAS Institute, Inc., Cary, NC), and a P < 0.05 was considered statistically significant.

## Results

### Ovarian Cyclicity Status

**Fig 2:**
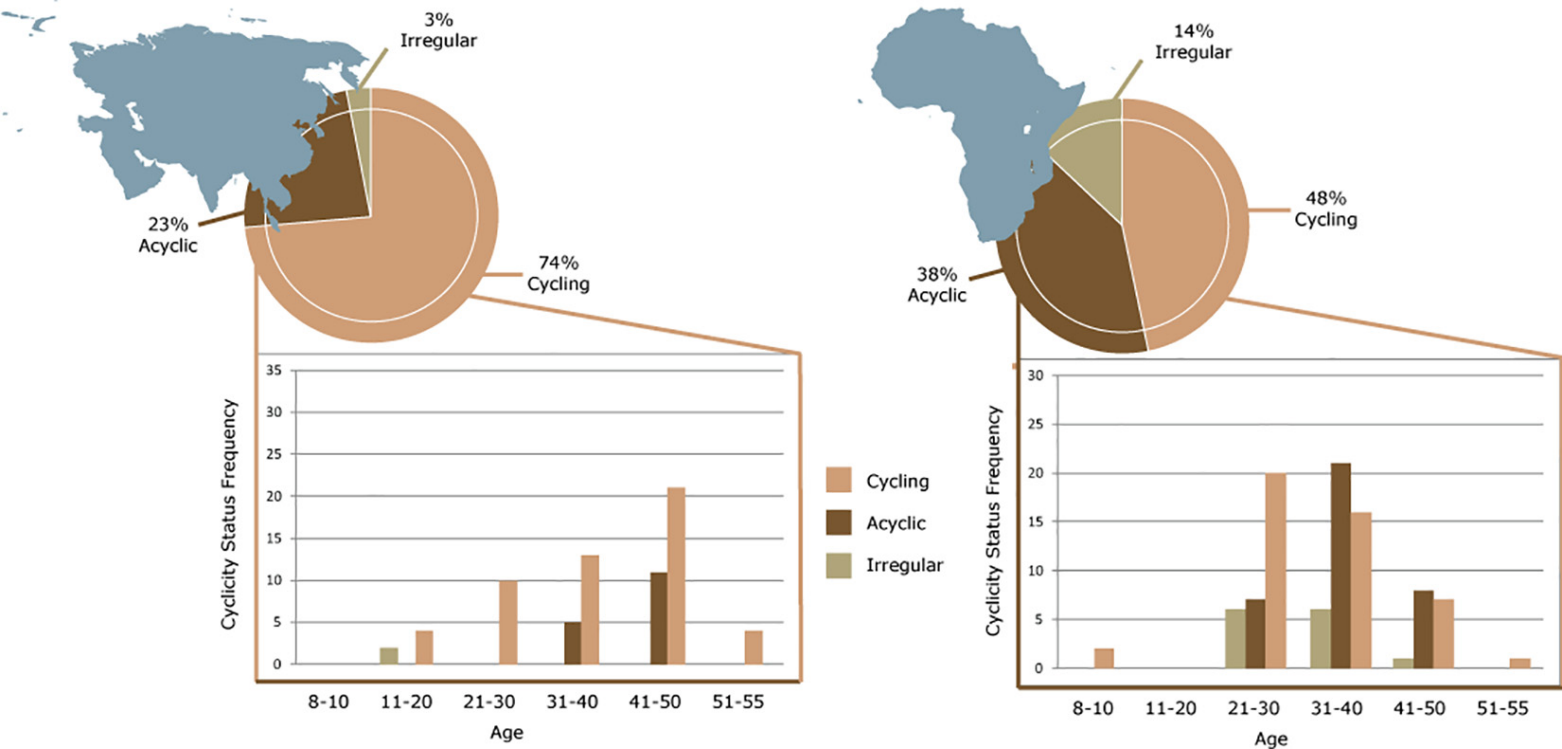
Proportion of normal cycling, irregular cycling and acyclic elephants in the study population for Asian (left) and African (right) elephants in pie charts, and below each pie chart is a further breakdown by age categories for each species.

Overall results of ovarian cycle status categorizations are presented in Table 2 and Fig 2. African elephants exhibited a higher rate of ovarian acyclicity as compared to Asian elephants (P < 0.05) (Table 2). Additionally, more African females experienced irregular ovarian cycles than did Asian females (P < 0.05). Overall rates of abnormal cycle activity (acyclic plus irregular cycles) were 26.7% for Asian and 51.6% for African elephants; a significant species difference (P < 0.05).

**Table 2:** Ovarian cyclicity status for female Asian and African elephants in the AZA population.

	Asian		African	
	N	Percent	N	Percent
Study population	71		95	
Cyclicity status				
Cycling	52	73.2^a^	46	48.4^b^
Acyclic	16	22.5^a^	36	37.9^b^
Irregular	3	4.2^a^	13	13.7^b^

^a,b^Superscripts denote differences (P < 0.05) in cyclicity status between species.

Within the study population, the youngest age that a female cycled was 15 years for Asian and 9 years for African, and the oldest age was 55 years for Asian and 54 years for African elephants. Reproductive cyclicity status as a function of age for both species is presented in Fig 2. Most acyclicity in post-pubertal Asian elephants was observed in females >36 years of age; only one Asian female less than 36 years of age was not cycling. For African elephants, acyclicity was observed in females as young as 27 years of age, with the majority of acyclicity found in the 31–50 year age categories. Overall, African elephant females were younger than Asians (33.1 ± 0.8 years vs. 38.5 ± 1.1 years, respectively), as was the mean age of acyclic African (35.8 ± 0.8 years) compared to acyclic Asian (43.6 ± 0.6 years) elephants (P < 0.05).

### Prolactin Status

Average prolactin concentrations were three times higher in African than Asian elephants, due to the high percentage (28%) of Africans with consistently High prolactin indicating hyperprolactinemia (P < 0.05) (Table 3). Even within the Low/Normal prolactin groups, concentrations were higher in African females because of previously characterized cyclic dynamics of prolactin secretion; i.e., increased during the follicular phase in African, but not in Asian elephants [10,19]. Prolactin levels were related to cyclicity status in African females (Table 4), with concentrations being higher in acyclic (24.02 ± 4.82 ng/ml) than cycling (10.75 ± 0.73 ng/ml) females, and in between for irregular cycling females (21.50 ± 2.34 ng/ml). While 44% (41/93) of African elephants with Low/Normal prolactin levels were cycling, only 12% (3/26) of African elephants with High prolactin levels were cycling normally. These three differed from acyclic hyperprolactinemic females in that their prolactin was elevated during the follicular phase only, similar to normal cycling elephants. Prolactin concentrations did not differ between cycling (6.50 ± 3.78 ng/ml) and acyclic (4.42 ± 2.87 ng/ml) Asian elephants (P > 0.05) (Table 5). In fact, only two female Asian elephants had a mean prolactin concentration over 14 ng/ml; these were both normal cycling, but older (~50 years) (Tables 3, 4 and 5).

**Table 3:** Mean (± SEM) and mean range of prolactin concentrations in Asian and African elephants with Low/Normal or High prolactin. Calculated cut-offs were < or ≥ 14 ng/ml for Asian, and < or ≥ 18 ng/ml for African elephants.

	Asian		African	
Prolactin Status	N	ng/ml	N	ng/ml
Low/Normal	73	6.71 ± 0.48^a^ (1.59–13.85)	67	9.65 ± 0.45^b^ (2.55–17.57)
High	2	21.26, 24.14	26	36.83 ± 4.80 (19.08–105.24)
All	75	6.98 ± 0.48^a^	93	22.75 ± 3.06^b^

^a,b^Superscripts denote differences (P < 0.05) in prolactin concentrations between species.

**Table 4:** Distribution/frequency (% of total) of Low/Normal (<18 ng/ml) and High (≥18 ng/ml) prolactin concentrations by cyclicity status for African elephants.

	Cyclicity Status			
Prolactin status	Irregular	Acyclic	Cycling	Total
Low/Normal	9 (10.0)	17 (18.3)	41 (44.1)	67
High	4 (4.3)	19 (20.4)	3 (3.2)	26
Total	13	36	44	93

**Table 5:** Distribution/frequency (% of total) of Low/Normal (<14 ng/ml) and High (≥14 ng/ml) prolactin concentrations by cyclicity status for Asian elephants.

	Cyclicity Status			
Prolactin status	Irregular	Acyclic	Cycling	Total
Low/Normal	1 (1.4)	16 (22.5)	50 (70.4)	67
High	2 (2.8)	0 (0.0)	2 (2.8)	4
Total	3	16	52	71

### Factors Associated with Cyclicity

Univariate modeling was performed on management and housing factors in relation to acyclicity for African elephants (Table 6), and the following were significant (P < 0.05): Age (positive), Percent Time with Restricted Physical Access (positive), and Enrichment Diversity (negative). Two other factors were nearly significant: Outdoor Space Experience (P = 0.0537; negative) and Percent Time Housed Separately (P = 0.0549; positive). Descriptive statistics for variables found to be significant in the univariate analysis are shown in Table 7.

**Table 6:** Univariate assessment of ovarian acyclicity using logistic regression. OR: Odds Ratio.

Hyp^1^	Variables	Reference	N	Beta	OR	*P* value
	*Demographics*^2^					
+	Age		82	0.086	1.090	0.026*
-	Origin	ref = Wild	76			
		Captive	6	-0.495	0.609	0.581
	*Space*^3^					
-	Space Experience		81	-0.004	0.996	0.279
+	Relative Space Experience Change		81	0.836	2.306	0.222
-	Space Experience per Elephant		81	-0.018	0.982	0.253
+	Space Experience Indoors		73	0.014	1.014	0.896
-	Space Experience Outdoors		81	-0.016	0.984	0.054^
-	Space Experience In/Out Choice		81	-0.001	0.999	0.761
+	Percent Time Indoors		81	0.001	1.001	0.926
-	Percent Time In/Out Choice		81	-0.012	0.988	0.303
-	Percent Time Outdoors		81	0.007	1.007	0.423
	*Social*^3^					
-	Herd		81	-0.065	0.937	0.457
-	Animal Contact		81	-0.054	0.948	0.620
-	Social Group Count		81	-0.033	0.968	0.374
-	Social Group Contact		81	-0.071	0.931	0.251
-	Social Experience		81	-0.325	0.723	0.089^
+	Relative Social Experience Change		81	0.278	1.321	0.747
+	Percent Time Housed Separately		81	0.022	1.022	0.055^
+	Percent Time Housed Separately with Restricted Physical Access		40	0.025	1.025	0.016*
-	Percent Time with Juveniles		81	-0.009	0.991	0.370
-	Percent Time Mixed Sex		81	-0.012	0.988	0.149^
	*Feeding*^4^					
+	Feed Total		79	0.035	1.035	0.653
-	Feeding Diversity		79	-1.542	0.214	0.100^
-	Alternative Feeding Methods		79	-1.335	0.263	0.270
+	Feeding: Spread		79	1.711	5.536	0.417
	*Management*^4^					
-	Enrichment Diversity		79	-3.394	0.034	0.016*
+	Enrichment Program		79	0.226	1.254	0.401
-	Exercise Diversity		79	-0.412	0.662	0.301

^1^Hypothesized direction of effect.

*P < 0.05

^P < 0.15; threshold for inclusion in multi-variable modeling.

See

^2^Prado-Oviedo et al. [45]

^3^Meehan et al. [46] and

^4^Greco et al. [47] for variable definitions and descriptions.

**Table 7:** Descriptive statistics for variables that were found to be significant in the cyclicity analyses for African elephants.

Variable	N	Mean	SEM	Minimum	Q1	Median	Q3	Maximum
Age^1^	82	33.1	0.8	8.0	29.0	32.5	36.0	52.0
Percent Time with Restricted Physical Access^2^	40	32.2	5.1	0.0	2.2	26.2	51.9	100.0
Enrichment Diversity^3^	79	2.9	0.0	2.5	2.7	2.8	3.0	3.3

See

^1^Prado-Oviedo et al. [45]

^2^Meehan et al. [46] and

^3^Greco et al. [47] for variable definitions and descriptions.

### Multi-variable Model

Multi-variable logistic predictive modeling found that the combination of Age and Enrichment Diversity to be most predictive of whether a female elephant would be acyclic, and the Odds Ratios for each significant variable are presented in Table 8.

**Table 8:** Multi-variable assessment of cyclicity in African female elephants using repeated measures logistic regression. OR: Odds Ratio.

Variables	Beta	OR	*P* value
Age^1^	0.08	1.09	0.0137
Enrichment Diversity^2^	-3.40	0.03	0.0222

See

^1^Prado-Oviedo et al. [45] and

^2^Greco et al. [47] for variable definitions and descriptions.

**Fig 3:**
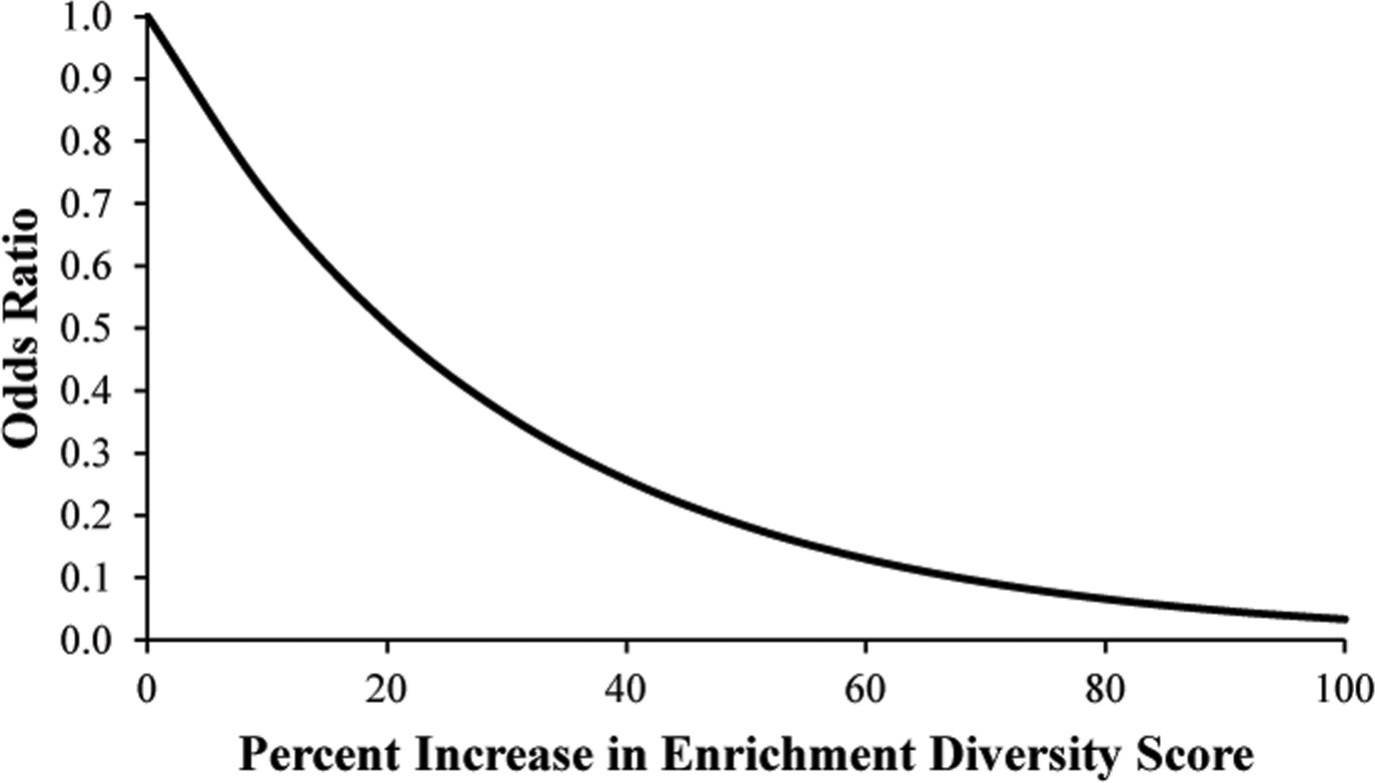
Odds of acyclicity decreases as Enrichment Diversity score increases. **Grey line represents 15-year-old elephant; black line represents a 30-year-old elephant.** See Greco et al. [47] for Enrichment Diversity Score definition and description.

Odds ratios show the effects of each variable conditional on the other variables; however it is illustrative to think about the effect each independent variable on the probability of an outcome separately. How this odds ratio associates Enrichment Diversity Score with cyclicity outcomes is illustrated using population-level descriptive statistics. So, for example, a 30-year-old elephant with an Enrichment Diversity Score of 2.7 (1^st^ quartile) has 0.0011 odds of acyclicity, while a 30-year-old elephant with an Enrichment Diversity score of 3.0 (3^rd^ quartile) has odds of 0.0004 for not cycling, such that an 10% increase in ED score is associated with a 64% decrease in odds of not cycling (Fig 3).

Age also was a significant factor in the model (Table 8). This relationship was non-linear and illustrated by maintaining Enrichment Diversity constant while looking at the change in odds for acyclicity with an increase in age. As such, a 30-year-old elephant at a zoo with an Enrichment Diversity score at the population average has odds of 0.0006 of being acyclic, whereas a 40-year-old elephant with the same Enrichment Diversity has odds of 0.0014 of being acyclic; as such, a 10-year increase in age was associated with a 122.5% increase in the odds of acyclicity.

### Factors Associated with Prolactin

Univariate modeling results for the prolactin data are shown in Table 9, which found several variables to be significant for the chance a female would be hyperprolactinemic (P < 0.05), both positive (Space Experience, Percent Time Outdoors, Social Group Count, Social Group Contact, Social Experience, Percent Time with Juveniles, Percent Time with Mixed Sex, Percent Independent Time) and negative (Percent Time Indoors, Alternate Feeding Methods, Enrichment Diversity). Other factors were nearly significant at the univariate level: Age (P = 0.0685; positive), Space Experience per Elephant (P = 0.0584; positive), Animal Contact (P = 0.0592; positive), Percent Time Managed (P = 0.0624; negative) and Exercise Diversity (P = 0.0624; negative). Descriptive statistics for variables found to be significant in the univariate analysis are shown in Table 10.

**Table 9:** Univariate assessment of hyperprolactinemia in African female elephants using logistic regression. OR: Odds Ratio.

Hyp^1^	Variables	Reference	N	Beta	OR	*P* value
	*Demographic*^2^					
+	Age		93	0.074	1.077	0.069^
-	Origin	ref = Wild	64			
		Captive	29	-0.283	0.754	0.581
	*Space*^3^					
-	Space Experience		91	0.010	1.010	0.006*
+	Relative Space Experience Change		91	0.096	1.101	0.869
-	Space Experience per Elephant		91	0.026	1.026	0.058^
	Space Experience Indoors		83	-0.043	0.958	0.737
+	Space Experience Outdoors		91	0.005	1.005	0.085^
+	Space Experience with In/Out Choice		91	-0.004	0.996	0.615
-	Percent Time Indoors		91	-0.024	0.976	0.034*
+	Percent Time Mixed Space		91	-0.024	0.976	0.095^
+	Percent Time Outdoors		91	0.037	1.038	0.002*
	*Social*^3^					
-	Herd Size		91	0.110	1.116	0.176
-	Animal Contact		91	0.201	1.223	0.059^
-	Social Group Count		91	0.060	1.062	0.025*
-	Social Group Contact		91	0.141	1.151	0.012*
-	Social Experience		91	0.305	1.357	0.011*
+	Relative Social Experience Change		91	0.897	2.452	0.195
+	Percent Time Alone		91	-0.014	0.986	0.272
+	Percent Time with Restricted Physical		45	0.005	1.005	0.670
	*Access*^3^					
+	Percent Time with Juveniles (<7 years old)		91	0.016	1.016	0.045*
+	Percent Time with Mixed Sex		91	0.022	1.022	0.017*
	*Feeding*^4^					
+	Feed Total		89	-0.029	0.971	0.684
-	Feeding Diversity		89	-1.432	0.239	0.212
-	Alternative Feeding Methods		91	-2.283	0.102	0.047*
-	Feeding: Spread		89	3.519	33.751	0.078^
	*Training*^4^					
+	Percent Time Spent Managed		82	-0.025	0.975	0.062^
-	Percent Independent Time		88	0.025	1.025	0.034*
	*Exercise and Enrichment*^4^					
-	Exercise Diversity		89	-0.790	0.454	0.062^
-	Enrichment Diversity		89	-4.039	0.018	0.005*
-	Enrichment Program		89	0.337	1.401	0.274

^1^Hypothesized direction of effect

*P < 0.05

^P < 0.15; threshold for inclusion in multi-variable modeling.

See

^2^Prado-Oviedo et al. [45]

^3^Meehan et al. [46] and

^4^Greco et al. [47] for variable definitions and descriptions.

**Table 10:** Descriptive statistics for variables that were found to be significant in the hyperprolactinemia analyses for African elephants.

Variable^1^	N	Mean	SEM	Min	Q1	Median	Q3	Maximum
Age	93	33.3	0.7	10.0	29.0	33.0	37.0	52.0
Enrichment Diversity	89	2.8	0.0	2.5	2.7	2.8	3.0	3.3
Alternative Feeding Methods	91	0.4	0.0	0.0	0.1	0.2	0.4	0.5
Space Experience (per 500 ft^2^)	91	61.2	6.4	4.9	18.8	35.2	86.8	295.1
Percent Time Indoors	91	30.6	2.4	0.0	11.3	25.4	52.9	71.5
Percent Time Outdoors	91	53.7	2.6	4.6	36.7	48.4	75.7	100.0
Social Group Count	91	7.4	0.9	1.0	3.0	5.0	10.0	43.0
Social Group Contact	91	3.7	0.5	1.0	1.0	2.0	4.0	30.0
Social Experience	91	2.8	0.2	1.1	2.0	2.8	3.0	11.2
Percent Time with Mixed Sex	91	13.84	3.09	0.0	0.0	0.0	0.0	100.0
Percent Time with Juveniles (<7 years old)	91	12.94	3.18	0.0	0.0	0.0	0.0	100.0
Percent Time Independent	88	50.94	2.3	0.0	23.3	50.0	66.4	87.0

^1^See Prado-Oviedo et al. [45], Meehan et al. [46] and Greco et al. [47] for variable definitions and descriptions

### Multi-variable Model

The multi-variable logistic predictive model found that the combination of Age, Enrichment Diversity, Alternate Feeding Methods and Social Group Contact was the most predictive with regards to high prolactin levels (Table 11).

**Table 11:** Multi-variable assessment of hyperprolactinemia (≥18 ng/ml prolactin) in African female elephants using repeated measures logistic regression. OR: Odds Ratio.

Variables	Beta	OR	P value
Age^1^	0.117	1.12	0.0073
Enrichment Diversity^2^	-3.670	0.03	0.0135
Alternate Feeding Methods^2^	-2.812	0.06	0.0438
Social Group Contact^3^	0.166	1.18	0.0010

See

^1^Prado-Oviedo et al. [45]

^2^Greco et al. [47] and

^3^Meehan et al. [46] for variable definitions and descriptions.

**Fig 4:**
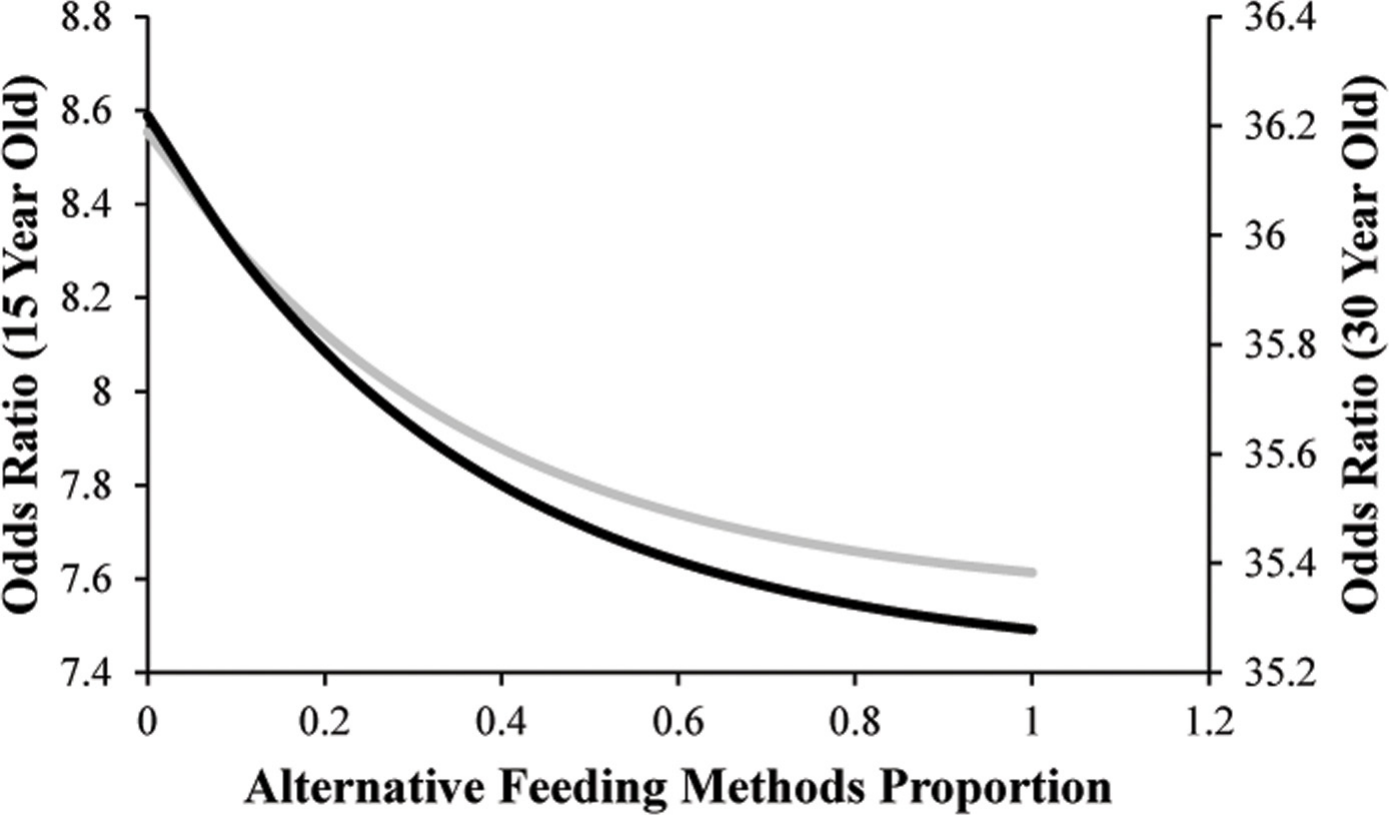
Odds of hyperprolactinemia for a 15-year-old (grey) and a 30-year-old (black) elephant with average Enrichment Diversity and Social Group Contact decreases as Alternative Feeding Methods proportion increases. See Meehan et al. [45] and Greco et al. [47] for variable definitions and descriptions.

While these models represent the conditional effects of the significant variables on an elephant’s risk for being hyperprolactinemic, the relative effect size of each individual variable can be interpreted through the associated Odds Ratio. The Odds Ratio for Enrichment Diversity score was 0.03, which indicates the same relationship with odds of hyperprolactinemia as for acyclicity. In addition, increased use of Alternate Feeding Methods, such as hanging food, hiding food or putting food in devices designed to increase the difficulty with which food is accessed, was associated with reduced odds of hyperprolactinemia. To illustrate this effect, we examined the change in odds of an elephant being hyperprolactinemic that would hypothetically result from an increase in Alternate Feeding Methods score when all other variables are held constant. So, for a 15-year-old elephant at a zoo with average Enrichment Diversity and Social Group Contact, an increase in Alternative Feeding Methods score from 20% to 50% (medial to 3^rd^ quartile) is associated with a 32.4% decrease in the risk of being hyperprolactinemic. A 30-year-old elephant with the same Enrichment Diversity and Social Group contact has the same 32.4% decrease in odds of becoming hyperprolactinemic, but the magnitude is much more pronounced than in the 15-year-old, because the basal odds of hyperprolactinemia are elevated in an older elephant (Fig 4).

The factor found to be most strongly associated with increased risk of hyperprolactinemia was Social Group Contact, which is a measure of the number of different social groups that an elephant spends any amount of time in [46]. The range of Social Group Contact scores for the study population was 1–28, and when Age (33 years), Enrichment Diversity (2.8 score) and Alternative Feeding Methods (0.4 score) were held constant, an increase from two social group contacts to four (median to 3^rd^ quartile) was associated with a 39% increase in the odds of a female being hyperprolactinemic.

## Discussion

Our findings demonstrate that reproductive dysfunction related to the pituitary-ovarian axis is prevalent in the North American population of managed African elephants at rates that not only raise concerns regarding the welfare of affected females, but also pose a threat to the long-term sustainability of the population [50]. Over half of African elephant females in the study exhibited irregular or a complete lack of ovarian cycle activity, compared to about a third of Asians, which is similar to rates observed over the past decade [4–6,11]. In addition, hyperprolactinemia was found in 53% of acyclic African elephants, which could be another indicator of compromised welfare in the zoo population. This percentage was intermediate to rates of 33% [10] and 71% [19] reported earlier, no doubt due to differences in population sampling among the three studies. The rate in this study should be more accurate because a greater proportion of the population was evaluated.

In normal cycling females, prolactin is folliculogenic and elevated during the nonluteal phase of the cycle [29,51]. Following this pattern, prolactin in African elephants is inversely related to progestogens, and phase-shifted by about 4 weeks [18,19]. However, too much prolactin can have negative consequences for normal ovarian function [20–22]. Prolactin is under inhibitory control from hypothalamic dopamine, and a similar mechanism is suggested for elephants based on observations of expected changes in prolactin secretion after administration of dopamine agonists and antagonists [5,28]. Hyperprolactinemia is often related to the development of prolactinomas, the most common pituitary tumor, as a result of reduced dopamine concentrations or sensitivity, or vasculature isolation that prevents dopamine from reaching the lactotrophs [26,27]. There are no data on the incidence of prolactin-secreting tumors in African elephants, as pituitary histopathology is rarely performed at necropsy, but this is a possibility and should be explored. Interestingly, in Asian elephants, prolactin concentrations are unvaried throughout the cycle and remain at baseline concentrations, representing a major species difference [10]. It may not be surprising, then, that ovarian cycle problems associated with abnormal prolactin secretion are common in African, but not Asian elephants [19]. Only two Asian elephants in this study had prolactin concentrations in excess of the high cutoff of 14 ng/ml, and both were cycling normally.

While the ‘symptom’ of ovarian inactivity in elephants clearly is physiological (i.e., baseline progestogen secretion), the ‘etiology’ likely involves both physiological and psychological mechanisms. Through the application of regression modeling, we identified a number of factors associated with increases in the odds of acyclicity in the African species. Similar analyses were not conducted with the Asian population due to lower rates of reproductive dysfunction. The strongest multi-variable models for African elephants included Age (negative) and Enrichment Diversity (positive) as important factors for cycling, whereas Age (positive), Enrichment Diversity (negative), Alternate Feeding Methods (negative) and Social Group Contact (positive) were predictors of hyperprolactinemia. Thus, social stability and enrichment appear to be important for promoting normal ovarian function and hormone status.

The relationship between age and ovarian acyclicity in African elephants was not unexpected, and suggests a form of reproductive senescence in elephants [52–54]. However, there is no clear evidence that elephants experience a true ‘menopause’ as in humans; i.e., the termination of ovarian cyclicity in midlife. The majority of Asian and nearly half of African elephants in the oldest age category (>40 years of age) were still cycling, including a number in their 50’s. In a large, comparative study of survival and reproduction between working elephants in Myanmar (>1,000 individuals) and pre-industrial humans (>5000), women experienced prolonged post-reproductive periods (i.e., menopause), whereas reductions in fertility in elephants paralleled declines in survivorship [54]. In other words, elephants retained the ability to reproduce until near death. A study of ovarian histology of female elephants culled in southern Africa further showed that although follicle reserves decline with age, the elephant ovary appears capable of supplying oocytes for ovulation right up to the time of death in most individuals [12]. Thus, termination of ovarian cyclicity in zoo elephants, especially in the younger age categories, is not a natural phenomonon. Age was also a significant factor in the multi-variable model predicting odds of hyperprolactinemia for African elephants, although such a relationship has not been well documented in other species (e.g., [55]). Given the fact that prolactin is a pleiotropic hormone with over 300 neuroendocrine functions, it is not clear why older females would be more likely to be hyperprolactinemic than younger ones. Altogether, while advancing age is a factor in the onset of reproductive dysfunction, the evidence suggests that conditions such as ovarian acyclicity and hyperprolactinemia are not simply artifacts of the aging process, and that housing, husbandry and management factors should be examined for their potential role in these outcomes in otherwise healthy elephants.

For example, the Enrichment Diversity factor was found to be associated with both ovarian cyclicity and hyperprolactinemia outcomes. Environmental enrichment is any technique designed to improve the biological functioning of a captive animal via modifications to its environment [56]. Enrichment Diversity is a measure of both the number of categories of enrichment utilized, and the frequency of their use within enrichment programs at each zoo [47]. The enhancing effects of enrichment on reproduction have been hypothesized and anecdotally reported for decades [57,58], but there are only a few experimental demonstrations of this effect in mammals: e.g., improved reproductive rates in laboratory mice living in enriched cages [59,60] and reduced rates of infertility in female farmed mink provided with manipulable objects [61]. In the latter case, increased activity and reduced aggression of enriched mink were suggested to be important mediators of this effect, perhaps via psychological mechanisms. Little research has been conducted on the welfare benefits of enrichment for elephants [62,63], and our study suggests that zoos should diversify the type and frequency of enrichment to increase novelty and unpredictability [47]. Adding varied types of enrichment to an animal’s enclosure may have psychological effects that include presenting biologically relevant information, providing greater contingency or control, and facilitating coping ability [64]. Enrichment typically stimulates or enhances active behaviors such as exploration, foraging, locomotion, self-care and play. Thus, when developing enrichment programs, knowledge of the species’ behavioral biology and natural history is essential to identify what environmental stimuli are needed for optimal psychological and physiological well-being [65]. To do so, it is necessary to understand how free-ranging elephants respond to environmental information within their natural habitats.

In many species, reproductive activity is timed to coincide with periods of increased resource availability [66]. Elephants in parts of African live in a tropical climate that varies between wet and dry periods, and some populations exhibit evidence of controlled timing of ovarian activity and anestrous around periods of seasonally optimal resource availability [52]. Thirty seven focal elephants in Northern Kenya exhibited decreased fecal progestogen concentrations in dry seasons, sometimes even ‘flatlining’. However, cycling resumed with the onset of the wet season such that ovulation was timed to the availability of high quality forage [52]. Interbirth intervals were also shorter in wet seasons and longer in dry periods [52,53]. Therefore, African elephant females appear to integrate information on known current and expected future conditions when reproducing [67]. Recent evidence indicates that the cues elephants use to monitor changing seasons may be more complex and diverse than solely a change in the quality of forage. Elephants alter their movement behavior (increase rate of forward movement, distance and area used) in response to seasonal changes in rainfall [68]. This change may occur days to weeks before any rain falls in the elephants’ location, and it is hypothesized that elephants rely on low-frequency (infrasonic) sounds produced by rainstorms as far as 300 km away to guide movements into habitats where browse is or will be available. Early flushes in savannah tree species that precede rainfall might also be tracked by elephants as a cue to move to new areas prior to actual rainfall [69]. Surface-water availability is another resource that drives local regulation of elephant population distribution [70]. Thus, there are many environmental cues that free-ranging African elephants may be using to adjust their behavior, cues that could influence ovarian status as a means to optimize the timing of reproduction. Hence, temporary acyclicity may be an evolutionary response of African elephant females to a real or perceived suboptimal environment for reproduction.

In a perhaps related finding, the majority of African elephant conceptions in the wild occurred after the maximum level of vegetation productivity [67]. Thus, African females consume most high quality vegetation before conception and may forego reproduction during seasons with poor ecological conditions [67]. The natural coupling of ovarian activity with environmental quality in female African elephants appears to be a flexible reproductive strategy, so in ecological terms, Africans elephants may be ‘capital breeders’ that produce offspring from resources acquired and stored prior to initiation of a reproductive bout [71]. For African elephants in zoos, it may be that low diversity of enrichment, including feeding options, signals an environment poor in resources, even though food and water are not limited. An unvarying, poorly enriched captive environment may be lacking in stimulus change, unpredictability or novelty that elephants need to trigger a hormonal state for reproduction. Alternate Feeding Methods, which is related to enrichment insofar as it is a measure of the frequency with which food is provided in a manner that supports the expression of foraging behavior, is also an explanatory factor for normal prolactin levels. This finding suggests that having foraging options is a component of elephants’ assessments of the quality of their environment in zoos, and that Enrichment Diversity and Alternative Feeding Methods, in combination, lead to a more dynamic environment to which elephants respond with optimal pituitary and/or ovarian function.

In examining social factors related to the chance a female would exhibit normal ovarian cycles, univariate analysis revealed that social isolation, either with or without the ability to interact with herd mates through a barrier, was associated with increased odds of being acyclic. While this variable was not included in the final multi-variable model, it is worth exploring given the relevance of sociality to elephant natural history. Elephants are highly social species, and in Africans, although association patterns in the wild are flexible, following fission-fusion dynamics, family groups are characterized by constant patterns of association, high frequencies of affiliative behavior (e.g., touching, rubbing, vocalizations) and coordinated movements and activities [72]. Kinship is important to elephant sociality, as females spend most of their lives together in the same group with first and second order maternal relatives [73]. For many social species, having and maintaining social relationships with conspecifics is critical to individual survival and well-being [74,75]. Among social species, the benefits of communal connections, even if individuals are unrelated, include reduced morbidity and mortality [76,77], better physiological function and fitness [78–81] and specifically reproduction [82,83]. Thus, social isolation may have a number of negative impacts on welfare, including effects on the reproductive potential of African elephants in zoos.

There was a social factor present in the multi-variable model predicting hyperprolactinemia as well: Social Group Contact was positively associated with hyperprolactinemia, meaning female elephants that spent time in a greater number of different social groups had higher odds of being hyperprolactinemic. In addition to its roles related to reproduction, prolactin has been considered a “stress hormone” because its release into circulation is thought to support adaptive physiological and behavioral responses to physical and emotional stressors [84]. In human females, hyperprolactinemia has been linked to life events involving social stressors, such as relationship conflict, a new job, death of a loved one and divorce [85–88], and our findings indicate that for female African elephants, not being in a stable social group may be a stressor that elicits an increased prolactin response. This relationship may be mediated by a disruption in social hierarchies, which can result in increases in aggression and decreases in-group cohesion. For example, a study of wild elephant herds disturbed by poaching in Tanzania found that unrelated females eventually formed new groups with stable hierarchies, but compared to undisturbed groups of related individuals, they had higher fecal glucocorticoid concentrations indicative of stress, and lower reproductive output [89]. For zoo elephants, social management practices that include dividing the herd into multiple social groups and housing elephants in a variety of social configurations may yield a more demanding social environment to which they respond with elevated prolactin production. And while increased levels of prolactin may help elephants cope with increased social demands, this may occur at the cost of normal reproductive function. Interestingly, a previous study of zoo elephant reproductive function found that African females who spent longer periods of time with the same social partners were *more likely* to be acyclic, although prolactin levels were not assessed [34]. Therefore, it is likely there are multiple features of the elephant’s social experience, including the number of groupings as well as the compatibility of social partners, which influence physiology. In other species, such stress responses can be modified by estrous cycle status; i.e., under estrogen vs. progesterone exposure, and not always in the same direction [29,90,91,92]; however, in the case of African elephants, all hyperprolactinemic elephants were acyclic, with low concentrations of progestagens [current study] and estrogens [8]. Additional research into prolactin’s role as an indicator of social stress in managed elephants, and studies investigating the impact of social management strategies on incidence of hyperprolactinemia are highly recommended, as are explorations of other markers of stress (glucocorticoids, behavior) as they pertain to pituitary-ovarian function.

In summary, there continues to be a species difference in the types and degree of reproductive problems, with African elephants apparently being more susceptible to factors affecting ovarian activity and prolactin secretion compared to Asian females. Overall rates of ovarian acyclicity for both species did not change between surveys conducted in 2009 [6,11] and our study; however, irregular cycles continued to increase, and may represent the transition from a cyclic to an acyclic state in some individuals. Prolonged acyclicity does not appear to occur in wild African elephants based on physiological studies that show females can cycle into their 50’s, although capacity does decline with age [32,33]. As such, the reproductive dysfunction we quantified is likely the result of management practices common to North American zoos. In fact, several non age-related factors, most notably those associated with sociality and environmental enrichment diversity, had significant influences on estrous cycle status and normal prolactin secretion. However, additional research is needed to further advance our understanding of the relationship between elephant management and reproductive outcomes, and it is unlikely that any one management factor is solely responsible for the current status of reproductive health among African elephant females. Rather, there probably are multiple etiologies, so it will be key to ascertain if problems are of physical or behavioral origin, and to determine the best approaches to amelioration. Continual longitudinal monitoring of ovarian and pituitary hormones can identify changes in reproductive potential of the zoo population, as well as provide insight into the welfare of individual elephants. Additionally, monitoring during management changes can lend evidence to our correlational data indicating that environmental enrichment, feeding methods and social management play a role in these outcomes. Clearly, we need to understand why so many females are not cycling normally; otherwise, the population collapse predicted for the U.S. will be inevitable [50]. Determining how social and environmental factors relate to prolactin secretion and reproductive status, and if husbandry changes could help improve these outcomes, is key to supporting optimal welfare and to the long-term population sustainability of zoo elephants.
